# Can molecular hydrogen supplementation reduce exercise-induced oxidative stress in healthy adults? A systematic review and meta-analysis

**DOI:** 10.3389/fnut.2024.1328705

**Published:** 2024-03-25

**Authors:** Yiting Li, Renjie Bing, Meng Liu, Zhangyuting Shang, Yan Huang, Kaixiang Zhou, Dapeng Bao, Junhong Zhou

**Affiliations:** ^1^College of Sports Medicine and Rehabilitation, Beijing Sport University, Beijing, China; ^2^College of Sports Coaching, Beijing Sport University, Beijing, China; ^3^College of Physical Education and Health Management, Chongqing University of Education, Chongqing, China; ^4^Shichahai Sports School, Beijing, China; ^5^College of Physical Education and Health Science, Chongqing Normal University, Chongqing, China; ^6^China Institute of Sport and Health Science, Beijing Sport University, Beijing, China; ^7^Hebrew SeniorLife Hinda and Arthur Marcus Institute for Aging Research, Harvard Medical School, Boston, MA, United States

**Keywords:** molecular hydrogen, oxidative stress, exercise, antioxidant capacity, healthy adults

## Abstract

**Objective:**

Exercise-induced oxidative stress affects multiple neurophysiological processes, diminishing the exercise performance. Hydrogen (H_2_) can selectively reduce excessive free radicals, but studies observed its “dual effects” on exercise-induced oxidative stress, that is, increasing or decreasing the oxidative stress. Therefore, we here conducted a systematic review and meta-analysis to quantitatively assess the influence of H_2_ on exercise-induced oxidative stress in healthy adults.

**Methods:**

We conducted a systematic review of publications across five databases. The following keywords were used for search strategy: [“hydrogen”[Mesh] or “molecular hydrogen” or “hydrogen rich water” or “hydrogen-rich water” or “hydrogen rich saline”] and [“Oxidative Stress”[Mesh] or “Antioxidative Stress” or “Oxidative Damage” or “Oxidative Injury” or “Oxidative Cleavage”] and [“randomized controlled trial”[Mesh] or “randomized” or “RCT”]. We included trials reporting the effects of H_2_ on exercise-induced oxidative stress and potential antioxidant capacity post-exercise in healthy adults. Additionally, subgroup analyses were conducted to explore how various elements of the intervention design affected those outcomes.

**Results:**

Six studies, encompassing seven experiments with a total of 76 participants, were included in our analysis. Among these studies, hydrogen-rich water, hydrogen bathing, and hydrogen-rich gas were three forms used in H_2_ administration. The H_2_ was applied in different timing, including before, during, or after exercise only, both before and after exercise, and repeatedly over days. Single-dose, multi-dose within 1 day and/or multiple-dose over days were implemented. It was observed that compared to placebo, the effects of H_2_ on oxidative stress (diacron-reactive oxygen metabolites, d-ROMs) was not significant (SMD = −0.01, 95%CI-0.42 to 0.39, *p* = 0.94). However, H_2_ induced greater improvement in antioxidant potential capacity (Biological Antioxidant Potential, BAP) (SMD = 0.29, 95% CI 0.04 to 0.54, *p* = 0.03) as compared to placebo. Subgroup analyses revealed that H_2_ supplementation showed greater improvement (SMD = 0.52, 95%CI 0.16 to 0.87, *p* = 0.02) in the antioxidant potential capacity of intermittent exercises than continuous exercise.

**Conclusion:**

H_2_ supplementation can help enhance antioxidant potential capacity in healthy adults, especially in intermittent exercise, but not directly diminish the levels of exercise-induced oxidative stress. Future studies with more rigorous design are needed to examine and confirm these findings.

**Systematic review registration:**

https://www.crd.york.ac.uk/PROSPERO/display_record.php?RecordID=364123, Identifier CRD42022364123.

## 1 Introduction

Physical exercise affects the redox state by activating the generation of free radicals, such as reactive oxygen species (ROS), and then the body’s antioxidant defense system up-regulates the endogenous antioxidant systems to maintain the redox state (1). Oxidative stress is characterized by an imbalance in the redox state of a cellular environment, which means the rate of ROS generation surpasses the rate of antioxidant defense (2). However, individuals may experience different degrees of oxidative stress at different intensity levels of physical exercise (3). For instance, the prolonged moderate-intensity exercise can help reduce the oxidative stress (1), and conversely, high-intensity exercise may induce a surge in ROS and reduce the production of antioxidant enzymes in the short term (4). The excessive levels of ROS production alter multiple neurophysiological processes, leading to the muscle fatigue, damage and inflammation (5). Therefore, the supplementation of exogenous antioxidants in high-intensity exercise may help eliminate the ROS (6, 7).

Different conventional antioxidant supplementations (e.g., vitamin C, vitamin E, resveratrol) have been implemented to reduce the ROS for athletes and other populations (8–11). However, it has been shown that supplementing these types of antioxidants may induce some side effects, such as the reduction in muscle contractile strength and exercise adaptation (12–15), which may potentially because these antioxidants may exacerbate redox dysregulation and induce over-removal of ROS, of which an appropriate level is actually helpful for the maintenance of physiological function, activation of signaling pathways and initiation of multiple biological processes (16, 17). Given the limitations of conventional antioxidants, a novel antioxidant that can effectively combat oxidative stress without interfering with other important functions is highly demanded.

Molecular hydrogen (H_2_) is a potential antioxidant to alleviate exercise-induced oxidative stress (16, 18, 19). H_2_ can selectively reduce ·OH and ·ONOO without reacting to other important signaling oxidants (e.g., H_2_O_2_) (20, 21). Notably, previous meta-analysis about the effects of hydrogen in oxidative stress have primarily focused on populations suffering from diseases (e.g., periodontal, diabetes mellitus type 2, hypercholesterolemia) (19, 22). Recently, more research efforts have been focused on exploring the effects of H_2_ on oxidative stress in healthy cohorts. However, research on the efficacy of H_2_ supplementation for oxidative stress shows dual effects. For instance, one study discovered that consuming hydrogen-rich water (HRW) for 3 days could lead to a decrease in Biological Antioxidant Potential (BAP) and diacron-reactive oxygen metabolites (d-ROMs) (23); while in another study, increasing the concentrations of d-ROMs was observed following one-week intake of HRW (24). In addition, it was observed that 2 weeks of continuous HRW intake did not produce any notable changes in the oxidative stress and antioxidant responses of participants (25). These conflicting results could be attributed to substantial variations in study designs, such as the types of exercises, the administration of H_2_ and the participant characteristics (e.g., training level). Consequently, there is a pressing need to more explicitly characterize the efficacy of H_2_ on the exercise-induced oxidative stress and antioxidant levels in humans. In addition, it remains to be further explored whether H_2_ promotes the establishment of redox state by eliminating oxidants or enhancing the antioxidant capacity.

This study aims to quantitatively evaluate the effects of H_2_ on exercise-induced oxidative stress in healthy adults through a systematic review and meta-analysis of peer-reviewed publications. The findings will offer novel and essential insights into the benefits of H_2_ intake for reducing oxidative stress related to exercise and will guide the meticulous design of future research in this area.

## 2 Methods

This study was conducted by the Preferred Reporting Items for Systematic Reviews and Meta-analyses (PRISMA) guidelines. This study was registered with PROSPERO (CRD42022364123).

### 2.1 Literature search

Two authors, RB and ML, independently conducted searches in the PubMed, Web of Science, Medline, SportDiscus, and PsycINFO databases, covering all records from their inception until February 10, 2024. The following Medical Subject Headings (MeSH) terms and keywords were used for search strategy: [“hydrogen”[Mesh] or “hydrogen rich water” or “hydrogen-rich water” or “hydrogen rich saline”] and [“Oxidative Stress”[Mesh] or “Antioxidative Stress” or “Oxidative Damage” or “Oxidative Injury” or “Oxidative Cleavage”] and [“randomized controlled trial”[Mesh] or “randomized” or “RCT”]. The comprehensive search strategy was shown in Supplementary Table S1. Manual searches of the reference lists of included studies and reviews were also performed.

Studies were selected based on the following inclusion criteria according to the PICOS principle: (1) Participants: participants were healthy adults with a mean age of 18 years or older, with no history of H_2_ supplementation or usage of performance-enhancing dietary supplements or medications; (2) Interventions: the intervention involved participants ingesting molecular hydrogen, with methods including inhalation of H_2_-rich gas (HRG), tube feeding of H_2_-rich solutions, intravenous injection of H_2_-rich saline, hydrogen bathing, consuming hydrogen-producing tablets, or drinking HRW; (3) Comparison: the presence of a control group receiving a placebo; (4) Outcomes: the measurement of outcomes included at least one indicator of oxidative stress (e.g., d-ROMs) or antioxidant potential (e.g., BAP) relate to exercise; (5) Study design: the study employed a randomized crossover design or a randomized controlled trial.

Articles were excluded for the following reasons: (1) the study was an animal trial; (2) it was impossible to obtain outcome data; (3) the publication was a review paper or a conference article; and (4) the publication was a duplicate of another already considered.

### 2.2 Data extraction, outcomes, and risk of bias assessment

Two independent reviewers (ML and RB) extracted relevant data from each included study (26). They compiled details from the publications, such as the study’s authors and year of publication, sample size, participant demographics (age, height, weight, sex, performance levels), methods of H_2_ administration, exercise interventions, and the specific outcome measures assessed. In instances of disagreement on any outcome measures, the two reviewers consulted with two additional authors, KZ and DB, and continued discussions until they reached a unanimous decision.

The primary outcomes were BAP (μM) and d-ROMs (UCARR). If the post-test values for these two indicators were unavailable, they were calculated using the provided formulas, where the correlation coefficient (Corr) was set at 0.5 (26, 27).

Meanpost=Meanpre+Meanchange

$$\mathrm{SDpo}=\frac{2\times \mathrm{Corr}\times \mathrm{SDpre}+\sqrt{\begin{array}{l} 4\times Corr^{2}\times \mathrm{SDpr}e^{2}-4\\ \times \left(\mathrm{SDpr}e^{2}-\mathrm{SDchang}e^{2}\right) \end{array}}}{2}$$

If any relevant data was missing, we tried to contact the corresponding author or other authors of that study via email to request it (26).

Two investigators (RB and ML) independently assessed the risk of bias in the included studies using the Cochrane Collaboration’s tool (28), which contains the following criteria: (1) selection bias; (2) performance bias; (3) detection bias; (4) attrition bias; (5) reporting bias; and (6) other sources of bias. Studies were categorized based on the risk of bias as follows: those with one or more items evaluated as having a high risk of bias were classified as high risk; studies were deemed low risk if all items were assessed and found to have a low risk of bias. Studies not meeting either criterion were considered to have a moderate risk of bias.

### 2.3 Data synthesis and grading the evidence

Data synthesis and analysis were performed using Revman 5.3 software (Cochrane Collaboration, Oxford, United Kingdom) and Stata version 16.0 (Stata Statistical Software, release 16; Stata Corp., College Station, TX, United States). Due to the small sample size of each study, the Hedges’g was determined by subtracting the mean difference in outcomes of the control group from that of the intervention group post-intervention. Effect sizes were classified as trivial (<0.2), small (0.2 ∼ 0.5), moderate (0.5 ∼ 0.8), or large (>0.8) (29). We assessed heterogeneity by measuring the inconsistency among the trial effects using the I^2^ statistic. This level of heterogeneity was categorized using the Cochrane Collaboration’s guidelines: trivial if less than 25%, low for 25 to 50%, moderate between 50 and 75%, and high if above 75% (30). Due to the small sample size of each study and small number of included studies, continuous data were performed with the DerSimonian-Laird random-effects model with Hartung-Knapp-Sidik-Jonkman variance correction (31, 32). Publication bias was evaluated with a funnel plot and Egger’s test. To identify potential sources of heterogeneity, subgroup analyses were conducted based on variables such as training level, the duration of H_2_ supplementation, and types of exercise. The Trim and Fill method was employed to perform sensitivity analysis of the results when significant asymmetry was found (33). All the statistical significance was set at *p* < 0.05.

Furthermore, we assessed the quality of evidence (e.g., very low, low, moderate, or high) for the outcomes by utilizing the Grading of Recommendations Assessment, Development and Evaluation (GRADE) approach. This method appraises the quality of evidence considering study limitations, imprecision, inconsistency, indirectness, and potential publication bias (34, 35). Study quality was downgraded for a high risk of bias (poor internal validity), inconsistency in data (significant heterogeneity), indirectness in relation to interventions and outcomes of interest, the imprecision of results (wide CIs), and the likelihood of publication bias (36).

## 3 Results

### 3.1 Study selection

**Figure 1:**
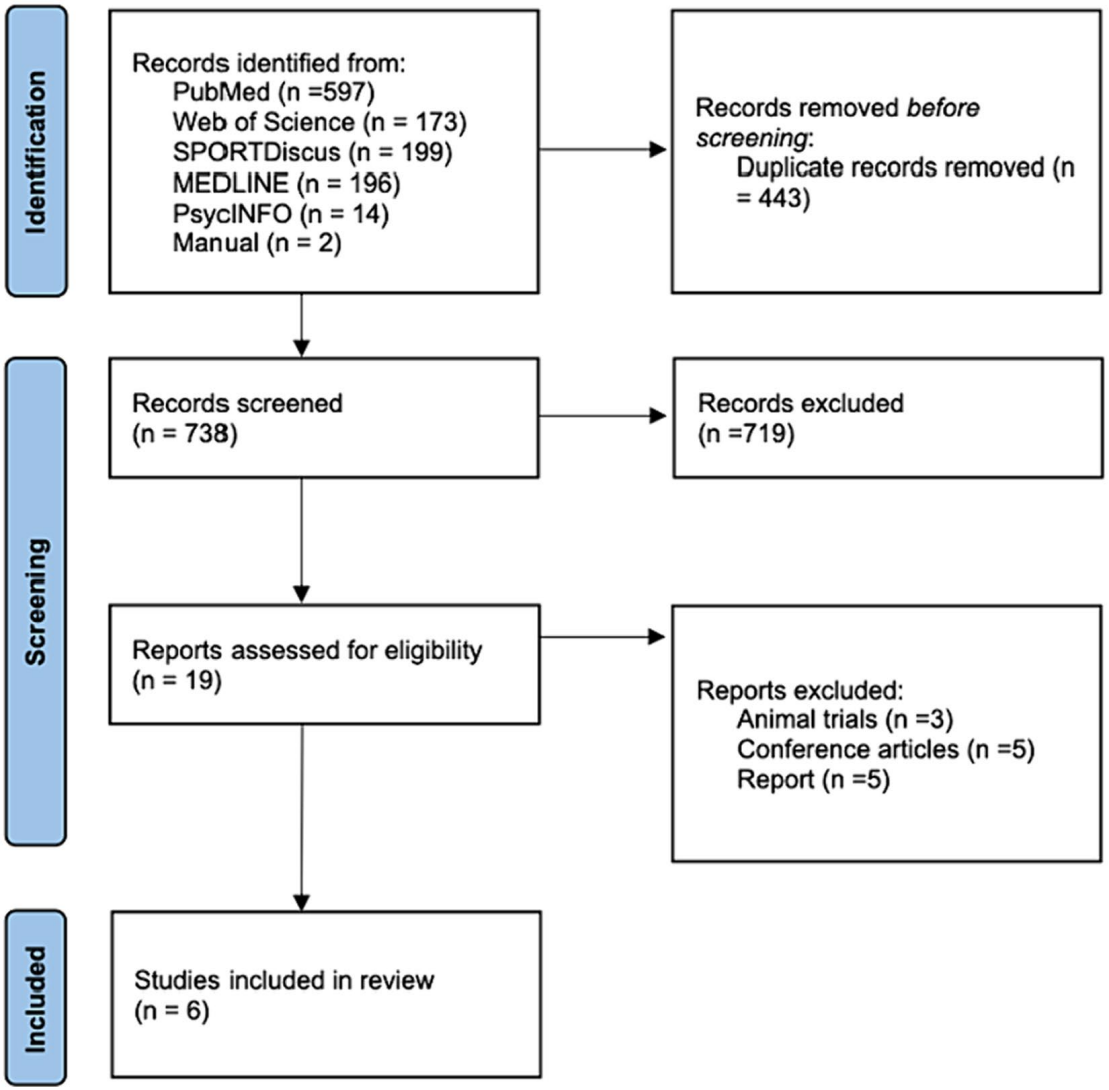
Study flowchart.

Figure 1 presents a flow diagram that summarizes the study identification and selection process. Our systematic search across various databases yielded a total of 1,181 records, broken down as follows: 597 from PubMed, 173 from Web of Science, 199 from SPORTDiscus, 196 from MEDLINE, 14 from PsycINFO, and 2 from manual searches. After the exclusion of 443 duplicate publications, we screened 738 articles. A further exclusion of 719 articles based on title and abstract reviews left us with 19 publications. Upon detailed evaluation of the full text of these 19 articles, 13 were excluded, ultimately resulting in 6 publications to be included in this systematic review and meta-analysis.

### 3.2 Participant characteristics

The sample size of the intervention groups ranged from 8 to 20 and a total of 76. One study recruited both men and women (25), and other studies only recruited male participants (23–25, 37–39). The mean ages of the participants ranged from 18.8 to 25 years. For the training status, one study focused on participants in recreationally trained soccer players (24); five studies focused on participants in untrained participants (23, 25, 37–39).

### 3.3 Intervention characteristics

The design of H_2_ administration, including the types of H_2_, timing to apply the H_2_, and its dosage was different across studies. Three types of H_2_ administration were used: three studies used drinking of HRW (23–25), one study used hydrogen bathing (39), and two utilized inhalation of HRG (37, 38). The timing of implementing/intaking H_2_ were: before (*n* = 2) (24, 25), during (*n* = 1) (38), or after exercise only (*n* = 2) (37, 39), both before and after exercise (*n* = 1) (23), and repeatedly daily over multiple days (*n* = 1) (25). For the dosage of intervention, four studies used single dose, one study used multiple doses within 1 day, and two studies used multiple doses over 3 days. The single dose included 500 mL HRW (*n* = 1) (25), 30 min (*n* = 1) (38) or 60 min (*n* = 1) (37) HRG, and 20 min hydrogen bathing (*n* = 1) (39). One study applied three doses of 500 mL HRG within 1 day (*n* = 1) (24). The multiple doses included continuous intake of 500 mL HRW for 3 days (*n* = 1) (23) or over 2 weeks (*n* = 1) (25). Details regarding the intervention characteristics were presented in Table 1.

**Table 1:** Characteristics of the included studies (*n* = 6).

Study	Design	Sample size	Participants’ characteristics					Methods of H_2_ administration	Exercise interventions	Outcome measures
			Age (yr)	Height (cm)	Weight (kg)	%F	People			
Aoki et al. (24)	RCD	10	20.9 (1.3)	172.0 (3.8)	67.1 (5.2)	0	Recreationally trained soccer players	HRW (H_2_ conc.:0.92 ~ 1.02 mg/L) Three 500 mL doses before exercise	Cycling for 30 min at 75% V̇O_2max_ and maximal knee extension exercise	Oxidative stress markers: d-ROMs→; BAP→ Others: BLA↓; CK→; Peak torque→; MF↓; MPF↓
Dobashi et al. (23)	RCT	8	19.4 (0.85)	174.3 (6.8)	67.6 (6.2)	0	Untrained physically active participants	HRW (Temp:4°C; H_2_ conc.:5.14 mg/L) 500 mL within 5 min before and after the exercise for 3 days	6 min repeated sprint cycling exercise	Oxidative stress markers: d-ROMs→; BAP→; BAP/d-ROMs ↑ Others: BLA→; CMJ→; MVIC→; Pmax; Pm for 10-s→
Shibayama et al. (37)	RCD	8	20.9 (0.3)	171.8 (1.6)	61.6 (1.5)	0	Untrained physically active participants	HRG (68% H_2_) 60 min after exercise	30 min Treadmill running (75%V̇O2max) and squat jump 5 × 10 rep.	Oxidative stress markers: d-ROMs→; BAP→; BAP/d-ROMs ↓ Others: CMJ↑; MVIC→; Pmax→; U8ER↓; CKa→; LDa→; White blood cells→; Pm→
Hori et al. (38)	RCD	12	21.8 (5.8)	174.5 (6.0)	67.7 (7.6)	0	Untrained healthy participants	HRG (1% H_2_) 30 min during exercise	Cycling for 30 min at 60% V̇O2peak	Oxidative stress markers: d-ROMs→; BAP→ Others: V̇CO_2_↑; V̇E↑;HRavg→;Vacetone↑; V̇O2 rest→; V̇CO_2_ rest→; V̇E rest→;Recovery HR →;Vacetone rest→; V̇O2peak↑
Hori et al. (Exp.1) (25)	RCD	9	19.9 (1.2)	169.6 (9.0)	63.7 (12.3)	33.3	Untrained university students	HRW (H_2_ conc.:4.3 mg/L) 500 mL doses at 35 min before exercise	Incremental cycling test to exhaustion	Oxidative stress markers: d-ROMs→; BAP→ Anti-fatigue: RPE→; BLA→ Others: Resting HR→; Pmax→; CDO→; RER→; V̇E→; HRmax→; V̇O2peak→
Hori et al. (Exp.2) (25)	RCT	20 (H:10/P:10)	20.3 (1.3)/20.4 (4.7)	175.5 (4.7)/172.6 (6.6)	69.9 (7.3)/67.8 (4.4)	0	Untrained university students	HRW (H_2_ conc.:5.9 mg/L) 500 mL on all weekdays for 2 weeks	Incremental cycling test to exhaustion	Oxidative stress markers: d-ROMs↓; BAP↑ Anti-fatigue: RPE→; BLA→ Others: Pmax→; CDO→; RER→; V̇E→; Resting HR→; HRmax→; V̇O2peak→;
Kawamura et al. (39)	RCD	9	25 (3)	174.0 (3.3)	66 (6.7)	0	Untrained physically active participants	Hydrogen Bathing Hydrogen water bathing for 20 min after exercise	30 min Downhill Running	Oxidative stress markers: d-ROMs→; BAP↑ Others: CK→; Blood lactate→

RCD, randomized crossover design; RCT, randomized controlled trial; BAP, biological antioxidant potential; d-ROMs, diacron-reactive oxygen metabolites; BLA, blood lactate; CK, creatine kinase; CKa, creatine kinase activity; CMJ, countermovement jump; CDO, carbon dioxide output; Exp., experiment; HRW, hydrogen-rich water; HRG, hydrogen-rich gas; HR, heart rate; HR_avg_, average heart rate; HR_max,_ maximum heart rate; LDa, lactate dehydrogenase activity; MF, median frequency; MPF, mean power frequency; MVIS, maximal voluntary isometric strength; RER, respiratory exchange ratio; P_m_, mean power; P_max_, maximum power; V̇E, ventilation volume; V̇O_2peak_, peak oxygen uptake; V̇O_2_, oxygen uptake; ↓, H_2_ significantly (*p* < 0.05) reduced the outcome compared to placebo; ↑, H_2_ significantly (*p* < 0.05) improved the outcome compared to placebo; →, no significant difference (*p* > 0.05) between H_2_ and placebo.

The studies incorporated two types of exercise, continuous and intermittent, to induce oxidative stress. Specifically, one study employed continuous incremental load exercise tests (25), four studies utilized continuous fixed-load exercise tests (24, 37–39), and one study conducted intermittent sprint exercises (23).

### 3.4 Outcome measurements

Six studies (seven experiments) used d-ROMs to assess oxidative stress levels (23–25, 37–39) and BAP (23–25, 37–39) to assess participants’ antioxidant potential capacity (Table 1). Four experiments conducted assessments immediately post-exercise (24, 25, 38); one at 16 h after exercise (23); one after post-exercise intaking of HRG for 60 min (37); and one after a 20 min post-exercise hydrogen bathing (39).

For d-ROMs, one study showed that molecular hydrogen supplementation significantly reduced d-ROMs as compared to the placebo (25), and the other six studies did not observe significant difference between molecular hydrogen supplementation and placebo (23–25, 37–39).

For BAP, two studies showed that molecular hydrogen supplementation significantly improved BAP compared to the placebo (25, 39), and the other five studies did not observe significant difference between the group of molecular hydrogen supplementation and placebo (23–25, 37, 38).

Two studies used BAP/d-ROMs (23, 37) to assess total antioxidant potential in serum. One study showed that molecular hydrogen supplementation significantly reduced BAP/d-ROMs compared to the placebo (23), and the other did not observe significant differences.

### 3.5 Risk of bias

**Figure 2:**
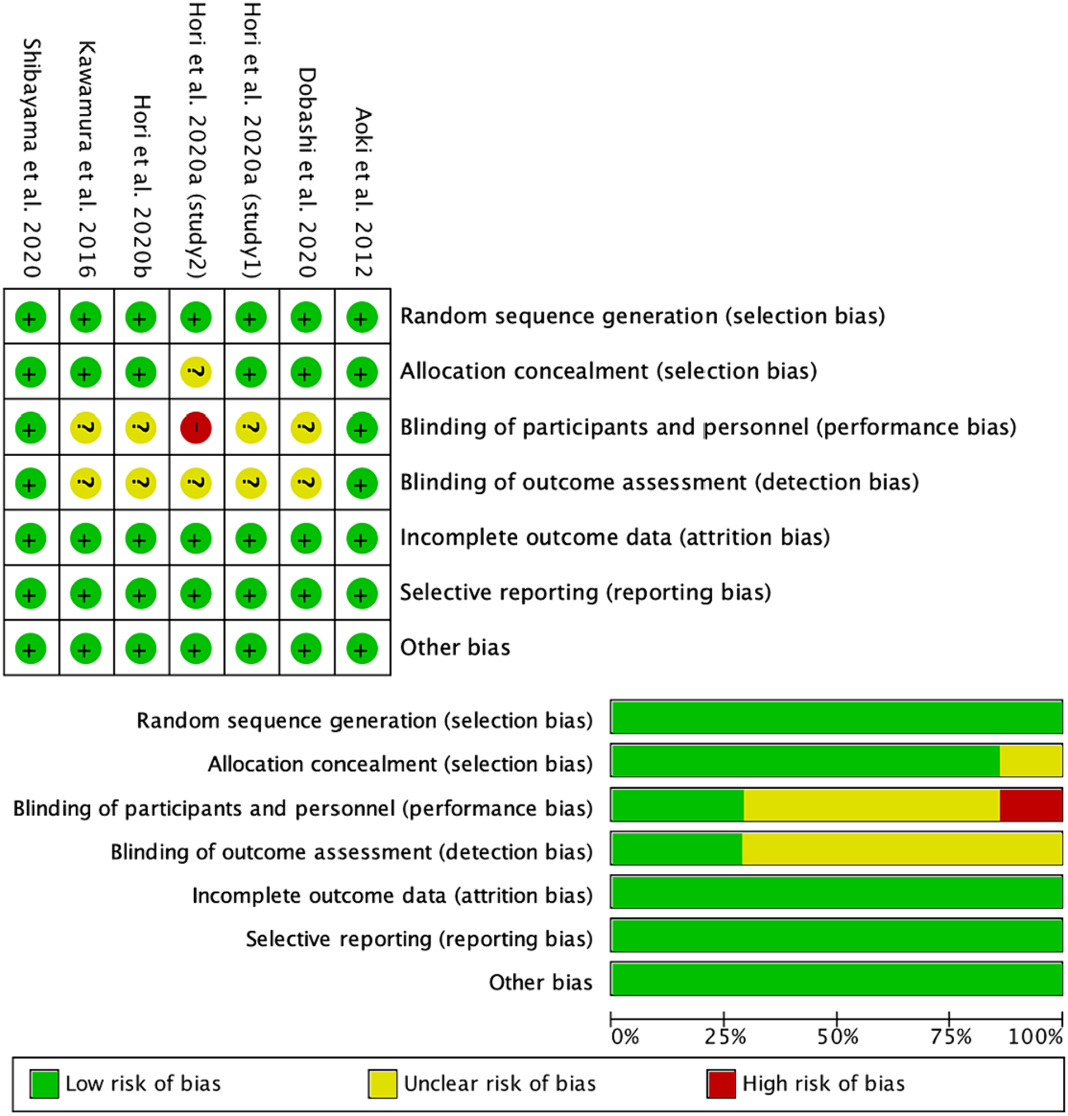
Risk of bias in the included studies.

Figure 2 displays the quality assessment results of the six included studies, comprising seven experiments. Four studies utilized a randomized crossover design, while the others adopted a randomized, double-blind, placebo-controlled design. The experiments were evaluated for potential bias and categorized as low, moderate, or high risk. Specifically, one study (17) was deemed to have a high risk of bias, four studies (16, 17, 28, 39) were classified as having a moderate risk, and the remainder were considered to have a low risk of bias.

### 3.6 Meta-analysis

We analyzed the effect of H_2_ on oxidative stress (d-ROMs) and its effect on antioxidant potential capacity (BAP). Because the two studies (23, 37) that included BAP/d-ROMs measured BAP/d-ROMs at different time points, we did not perform the analysis on this outcome.

Considering the observed heterogeneity, subgroup analyses were conducted for d-ROMs and BAP. These analyses compared the effects between different performance levels (untrained versus trained), varied periods of H_2_ implementation (single or multiple doses within a single day, and multiple doses extending beyond 1 day), and types of exercise (continuous versus intermittent).

#### 3.6.1 Effects of H_2_ on oxidative stress

**Figure 3:**
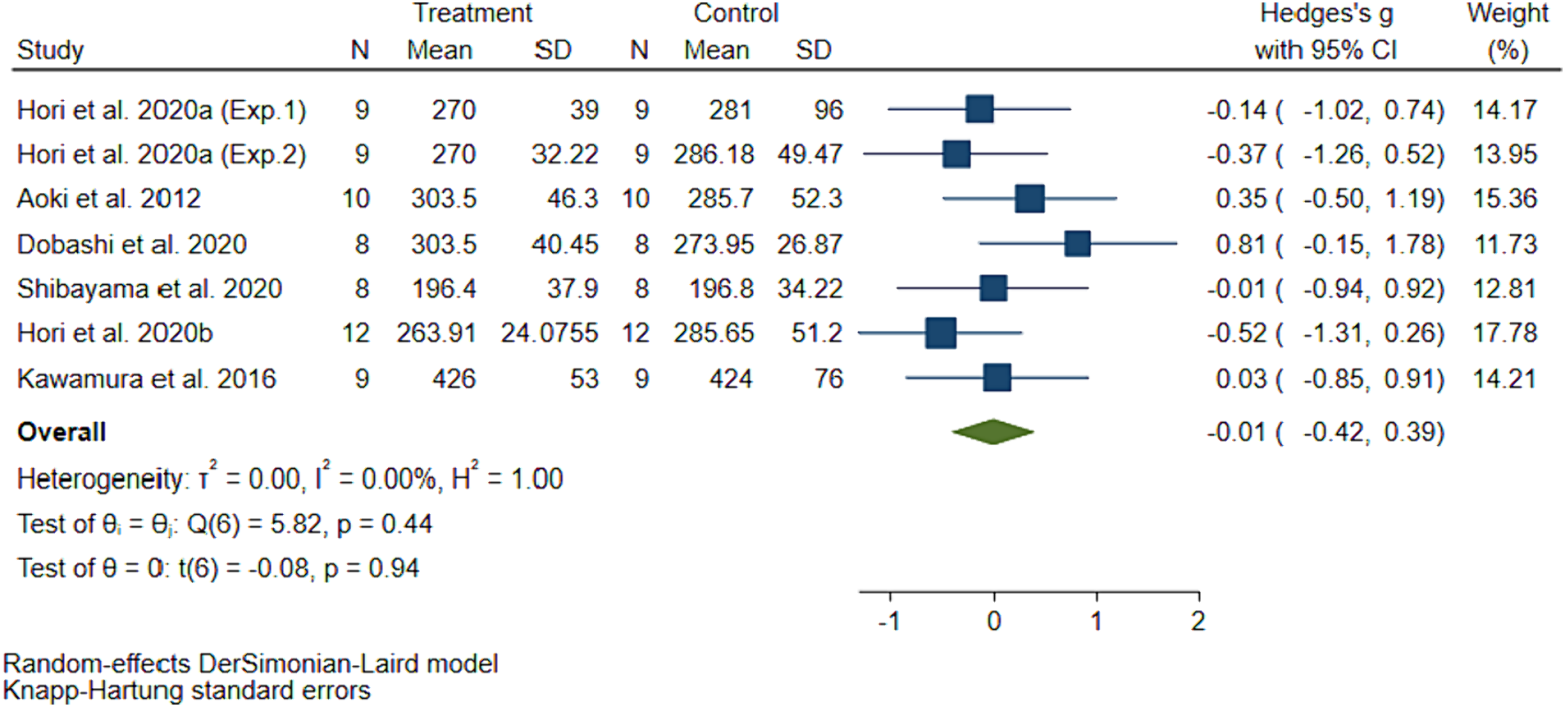
The pooled effect size of H_2_ intake on oxidative stress.

**Figure 4:**
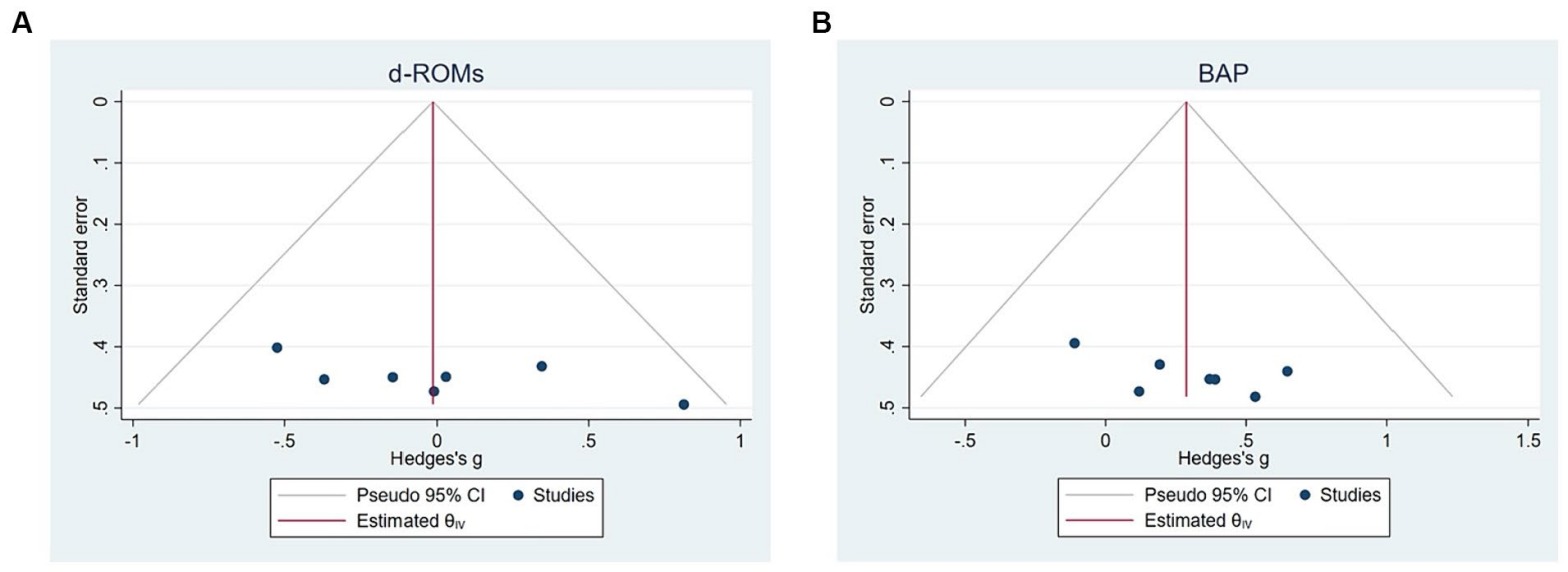
Funnel plots: **(A)** d-ROMs; **(B)** BAP.

The pooled ES of oxidative stress (d-ROMs) was not significant (SMD = −0.01, 95%CI-0.42 to 0.39, *p* = 0.94, Figure 3) and with low heterogeneity (I^2^ = 0%, *p* = 0.44). The assessment of publication bias using a funnel plot (Figure 4A) and Egger’s test (*t* = 1.62, *p* = 0.11) revealed no significant bias. Similarly, subgroup analyses showed no significant discrepancies (Table 2).

**Table 2:** Subgroup analysis results regarding the effects of H_2_ on anti-oxidative stress.

Outcomes	Variables	No. of experiments	Hedges’ g (95%CI)	*p* value	Test of heterogeneity		
					*χ^2^*	*p* value	*I^2^* (%)
d-ROMs	**Performance levels**						
	Untrained	6	−0.08 (−0.55, 0.39)	0.69	0	0.42	0.11
	Recreationally trained	1	0.35 (−0.50, 1.19)	-	0	-	-
	**Period of H**_**2**_ **implementation**						
	Single or multiple doses in 1 day	5	−0.08 (−0.49, 0.34)	0.63	0	0.58	0
	Multiple doses lasting more than 1 day	2	0.21 (−7.31, 7.72)	0.79	0.47	0.08	67.87
	**Exercise types**						
	Continuous exercises	4	−0.06 (−0.67, 0.54)	0.76	0	0.52	0
	Intermittent exercises	3	0.08 (−1.46, 1.61)	0.85	0.15	0.18	41.64

BAP	**Performance Levels**						
	Untrained	6	0.30 (−0.00, 0.61)	0.05	0	0.83	0
	Recreationally trained	1	0.19 (−0.65, 1.03)	-	0	-	-
	**Period of H**_**2**_ **implementation**						
	Single or multiple doses in 1 day	5	0.18 (−0.99, 0.44)	0.14	0	0.92	0
	Multiple doses lasting more than 1 day	2	0.59 (−0.13, 1.32)	0.06	0	0.86	0
	**Exercise types**						
	Continuous exercises	4	0.13 (−0.21, 0.47)	0.31	0	0.87	0
	Intermittent exercises	3	0.52 (0.16, 0.87)	0.02	0	0.91	0

#### 3.6.2 Effects of H_2_ on antioxidant potential capacity

**Figure 5:**
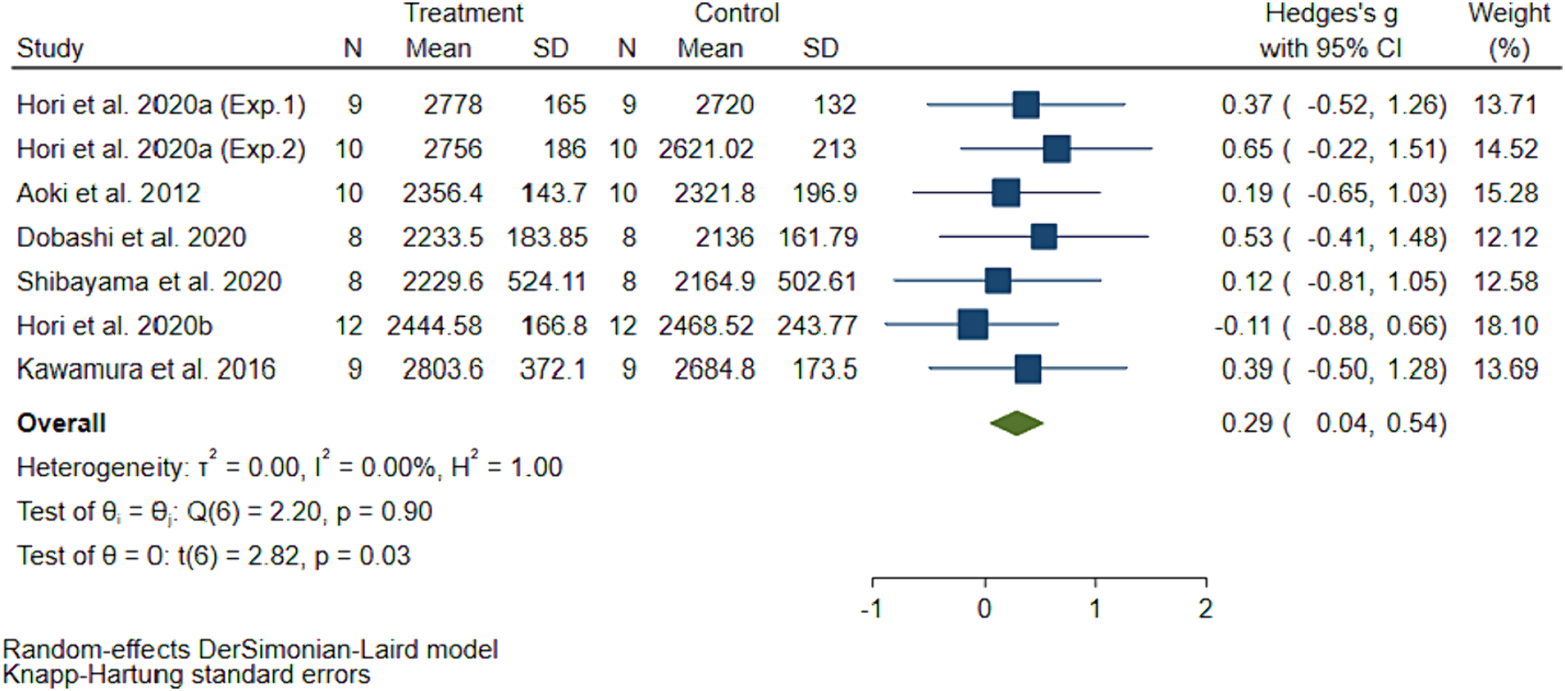
The pooled effect size of H_2_ intake on antioxidant potential capacity.

The pooled ES of antioxidant potential capacity (BAP) was significant (SMD = 0.29, 95% CI 0.04 to 0.54, *p* = 0.03, Figure 5) and with no heterogeneity (I^2^ = 0%, *p* = 0.90). The funnel plot (Figure 4B) and Egger’s test (*t* = 0.92, *p* = 0.36) indicated that there was no publication bias. The subgroup analysis demonstrated that molecular hydrogen supplementation showed greater improvement (SMD = 0.52, 95%CI 0.16 to 0.87, *p* = 0.02) in the antioxidant potential capacity of intermittent exercises (Table 2).

### 3.7 GRADE assessment

The quality of evidence for the outcomes has been assessed as moderate. For a detailed evaluation according to the GRADE framework, please refer to Supplementary Table S2.

## 4 Discussion

To the best of our knowledge, this systematic review and meta-analysis is the inaugural exploration into the impact of H_2_ supplementation on exercise-induced oxidative stress in healthy adults. The findings indicate that H_2_ supplementation holds potential as an effective means to enhance antioxidant capacity, although it does not seem to directly mitigate oxidative stress levels. Further, subgroup analysis has shown that the type of exercise influences the efficacy of H_2_ on enhancing antioxidant potential. The overall quality of the evidence gathered is moderate. Through quantitative analysis, our study contributes valuable insights into the capability of H_2_ supplements to diminish oxidative stress provoked by exercise.

### 4.1 Research gap

Based upon the results from animal studies only, previous studies have suggested the effects of hydrogen on the alleviation exercise-induced oxidative stress (2). However, such effects of H_2_ in humans remain unclear. In a recent meta-analysis, Zhou et al., observed differences in the alleviation of exercise-induced fatigue by hydrogen across different training levels and types of exercise. Taken together, such benefits of H_2_ may be associated with its antioxidative capacity (40), which, however, was not explicitly examined. Recent research efforts have been put to explore the effects of H_2_ on oxidative stress in humans (23, 37). However, varied or even controdictoray observations were reported (24, 37). This inconsistency may arise from the variance in the design of study protocol, and the potential “dual effects” of H_2_ on oxidative stress. Therefore, to bridge the current research gap in this field, we performed this work to comprehensively characterize the effects of H_2_ oxidative stress in humans, and more importantly, to explore the influences of the protocol parameters of implementing H_2_ on such effects.

No significant reduction in exercise-induced oxidative stress levels in healthy adults as induced by H_2_ supplementation was observed here. One possible reason is that H_2_ possesses a multifaceted role beyond merely scavenging ROS. Studies have suggested that H_2_ is capable of both neutralizing ROS and augmenting oxidative stress levels to maintain a balanced oxidative state (i.e., dual effects) (16). Such dual effect may induce bi-directional effects on the exercise performance. Specifically, the decrease of ROS can help alleviate fatigue but may affect the adaptation to the exercise load (41); while the increase of ROS may boost such adaptation to exercise, but may diminish the exercise performance (17, 42). Similarly, studies also showed the varied effects of H_2_ on the ROS related to disease (43, 44). For example, Honirayama et al., showed that inhalation of HRG increased urinary 8-hydroxy-2′deoxyguanine (8-OHdG) levels, a marker of DNA oxidation, in people with Parkinson’s disease (45); while Yoritaka et al., observed no significant increase of 8-OHdG after the inhalation of HRG with large dose (46). Therefore, the effects of H_2_ on ROS should be taken with cautious. Recent studies have provided preliminary evidence that such dual effects may be related to the dose of H_2_. For example, Murakami et al. observed that compared to 3 h of pretreatment of H_2_, lower doses (i.e., 1 h of pretreatment) of H_2_ can induce superoxide production (47), inducing greater level of ROS; while Xue et al. suggested that the HRW with higher doses (i.e., 0.82 mg/L) of hydrogen can significantly inhibit the activity of myeloperoxidase (MPO) than that of lower doses (i.e., 0.22 mg/L) (48). Future studies are thus highly demanded to explicitly characterize the relationship between the protocol of H_2_ supplementation and its effects on ROS, which will ultimately inform the appropriate design of interventions using H_2_ to alter the antioxidant capacity in humans.

It is observed that the hydrogen supplementation can significantly enhance the antioxidant capacity as captured by increased BAP levels after the inhalation of H_2_. This enhancement may be attributed to the activation of Nrf2 under oxidative stress conditions, leading to a rise in antioxidant enzymes such as superoxide dismutase and catalase. Previous research indicated that H_2_ reduced intracellular ROS accumulation in vitiligo specimens, concomitant with the enhancement of antioxidant enzyme activity (49). The subgroup analysis revealed that H_2_ is more effective in enhancing BAP level following HIIT. Compared to low-intensity continuous exercise, HIIT has been substantiated to generate a greater volume of free radicals, thereby inducing heightened oxidative stress (50). The effects of H_2_ on BAP level may be related to the amount of ROS produced, and the potentiating effect of hydrogen on antioxidant enzymes is influenced by the degree of oxidative stress. Therefore, the increased amount of oxidative stress in HIIT may augment the potentiating effect of hydrogen (49), suggesting the great promise of using H_2_ for the recovery from HIIT.

### 4.2 Limitation

It is important to acknowledge several limitations of this study. The number of included studies is still relatively small, limiting the power of evidence and the number of sub-group analyses. Due to the limited number of relevant studies, our research did not perform the sub-group analysis on several outcomes (e.g., BAP/d-ROMs). Additionally, our study did not conduct subgroup analyses between the modes of hydrogen gas administration (e.g., H_2_ dissolved in saline), limiting the discussion on the influences of the intaking mode of H_2_ on its antioxidant effects. Moreover, some of the included studies were not double-blinded, possibly leading to bias in the observations. Lastly, among the included studies, the longest duration of H_2_ intake was 14 days. This prevents us from examining the long-term effects of H_2_ on oxidative stress. To solidify the findings of this research and to uncover the long-term effects of H_2_ on exercise-induced oxidative stress, future studies should employ more rigorous designs and larger sample sizes. In addition, the inconsistent sampling time points may lead to variations in oxidative stress levels, thus the results of analysis should be concluded with caution.

## 5 Conclusion

H_2_ supplementation can help enhance potential antioxidant capacity in healthy adults but not via directly lower the levels of exercise-induced oxidative stress, especially in intermittent exercise.

## Data availability statement

The original contributions presented in the study are included in the article/Supplementary material, further inquiries can be directed to the corresponding authors.

## Author contributions

YL: Conceptualization, Methodology, Software, Writing – original draft. RB: Conceptualization, Formal analysis, Methodology, Software, Writing – original draft. ML: Data curation, Formal analysis, Writing – original draft. ZS: Data curation, Validation, Writing – original draft. YH: Supervision, Writing – review & editing. KZ: Conceptualization, Formal analysis, Supervision, Writing – review & editing. DB: Funding acquisition, Methodology, Supervision, Writing – review & editing. JZ: Supervision, Writing – review & editing.
