# Supplementary material for: Can molecular hydrogen supplementation reduce exercise-induced oxidative stress in healthy adults? A systematic review and meta-analysis
Source: Front Nutr. 2024 Mar 25;11:1328705. doi: 10.3389/fnut.2024.1328705 (PMC10999621; doi:10.3389/fnut.2024.1328705)
Supplement: Supplementary file 1 [file Table_1.DOCX]

Supplementary Material

# Supplementary Figures and Tables

## Supplementary Tables

**Supplementary Table 1.** Search Strategy

| Process | Keywords |
| --- | --- |
| # 1 | "hydrogen"[Mesh] OR "molecular hydrogen" OR "hydrogen rich water" OR "hydrogen-rich water" OR "hydrogen rich saline" OR "hydrogen-rich saline" OR "H_2_-dissolved water" OR "H_2_-infused water" OR "hydrogen gas" OR "hydrogen inhalation" OR "hydrogen bathing" |
| # 2 | "Oxidative Stresses"[Mesh] OR "Stress, Oxidative" OR "Antioxidative Stress" OR "Antioxidative Stresses" OR "Stress, Antioxidative" OR "Anti-oxidative Stress" OR "Anti oxidative Stress" OR "Anti-oxidative Stresses" OR "Oxidative Damage" OR "Oxidative Damages" OR "Oxidative Stress Injury" OR "Oxidative Stress Injuries" OR "Oxidative Injury" OR "Oxidative Injuries" OR "Oxidative Cleavage" OR "Oxidative Cleavages" OR " Oxidative and Nitrosative Stress" OR "Oxidative Nitrative Stress" OR "Oxidative Nitrative Stresses" OR "Nitro-Oxidative Stress" OR "Nitro Oxidative Stress" OR "Nitro-Oxidative Stresses" |
| # 3 | "randomized controlled trial" [Mesh] OR "randomized" OR "RCT" |
| # 4 | # 1 AND # 2 AND # 3 |

**Supplementary Table 2.** The quality of the evidence (GRADE)

| **Quality assessment** | | | | | | | **No of participants** | | **Effect** | **Quality** | **Importance** |
| --- | --- | --- | --- | --- | --- | --- | --- | --- | --- | --- | --- |
| **No of experiments** | **Design** | **Risk of bias** | **Inconsistency** | **Indirectness** | **Imprecision** | **Other considerations** | **H2** | **Placebo** | **Absolute** |  |  |
| **d-ROMs (Better indicated by lower values)** | | | | | | | | | | | |
| 7 | randomised trials | serious^1,2^ | no serious inconsistency | no serious indirectness | no serious imprecision | none | 76 | 76 | Hedge’s g 0.01 lower (0.42 lower to 0.39 higher) | ⊕⊕⊕⊝ | CRITICAL |
|  |  |  |  |  |  |  |  |  |  | MODERATE |  |
| **BAP (Better indicated by higher values)** | | | | | | | | | | | |
| 7 | randomised trials | serious^1,2^ | no serious inconsistency | no serious indirectness | no serious imprecision | none | 76 | 76 | Hedge’s g 0.29 higher (0.04 higher to 0.54 higher) | ⊕⊕⊕⊝ | CRITICAL |
|  |  |  |  |  |  |  |  |  |  | MODERATE |  |

^1^ Risk of performance bias
^2^ Risk of selective bias
